# Supplementary material for: Optical focusing inside scattering media with time-reversed ultrasound microbubble encoded light
Source: Nat Commun. 2015 Nov 24;6:8968. doi: 10.1038/ncomms9968 (PMC4673873; doi:10.1038/ncomms9968)
Supplement: Supplementary Information — Supplementary Figures 1-7, Supplementary Note 1, Supplementary Methods and Supplementary References [file ncomms9968-s1.pdf]

Supplementary Figures

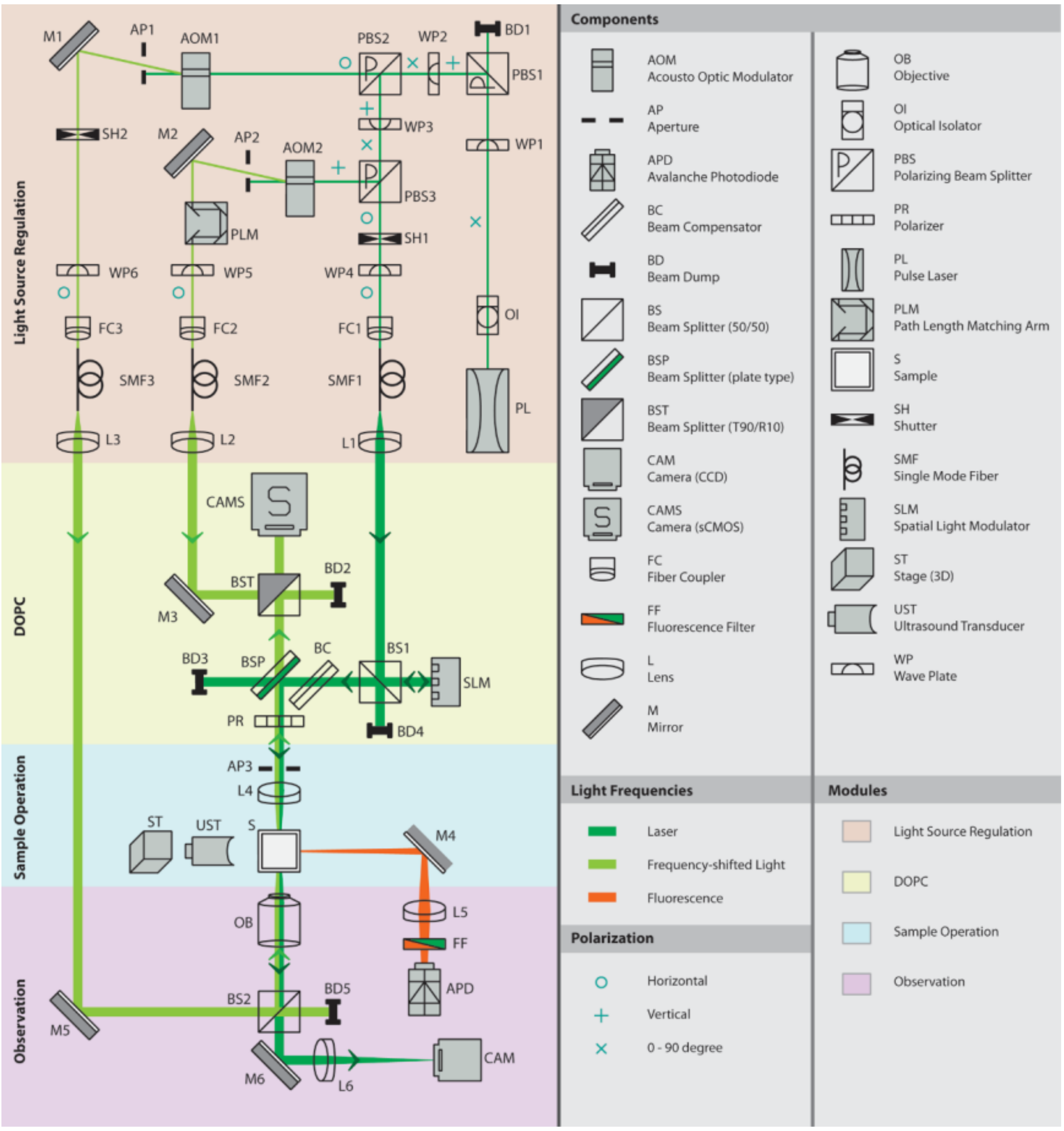

Supplementary Figure 1 | Optical setup schematic diagram. See Supplementary Methods for detailed description.

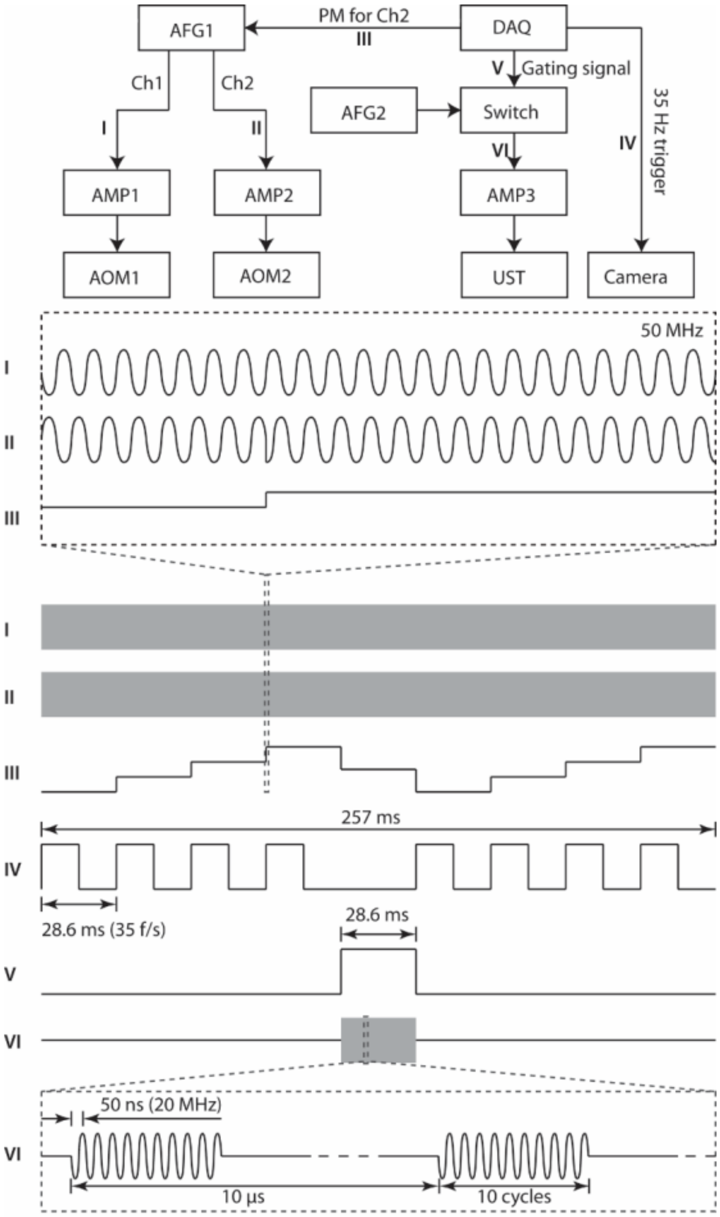

Supplementary Figure 2 | Electrical signal flow diagram. A function generator (AFG1) generates two 50 MHz continuous wave signals separately from two channels to drive the AOMs. A four-phase stepping digital holography method is used to capture the field of the light scattered from the sample. During DOPC recording, a data acquisition card (DAQ) generates a voltage stepping signal to modulate the phase of the signal from channel 2 of AFG1. At the same time, it outputs a synchronized trigger signal (35 Hz) to the camera of the DOPC system. After the first field is captured, the DAQ generates a gating signal to enable the ultrasound tone burst from the second function generator (AFG2). The phase recording process takes ~260 ms and the playback takes ~20 ms, leading to a total of ~280 ms for the TRUME process. Abbreviations: AFG – Arbitrary Function generator; AMP – Amplifier; AOM – Acousto-Optic Modulator; Ch – Channel; DAQ – Data Acquisition Card; PM – Phase modulation; UST – Ultrasound Transducer.

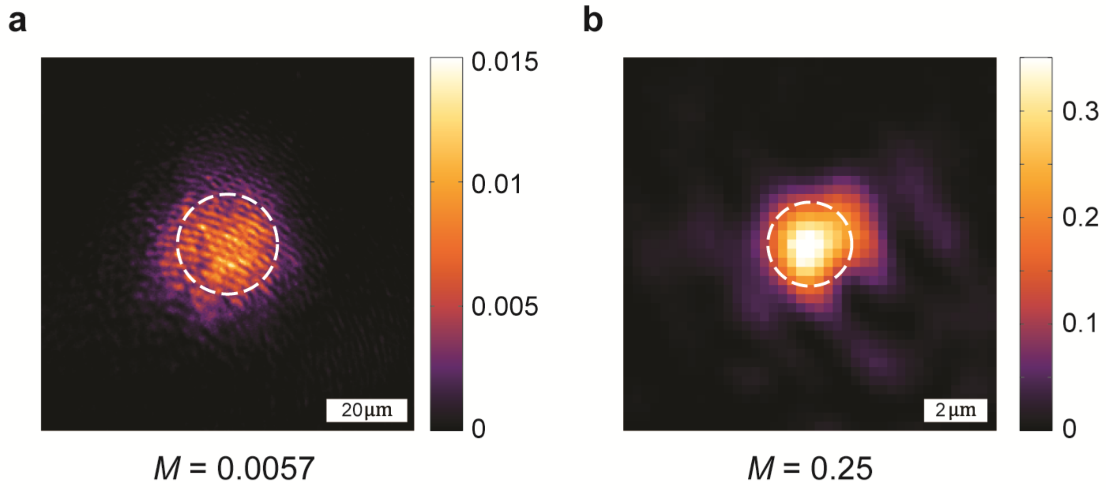

Supplementary Figure 3 | Experimentally measured modulation efficiency of direct ultrasound modulation and microbubble modulation. (a) Ultrasound modulation efficiency based on the holographical image of the ultrasound focus in clear water. (b) Measured microbubble modulation efficiency based on the field subtraction method of TRUME. The mean modulation efficiency M was calculated by averaging the modulation efficiency on each pixel over the ROI (circles), which is defined by the FWHM of the modulation efficiency across centre of each target in the horizontal direction.

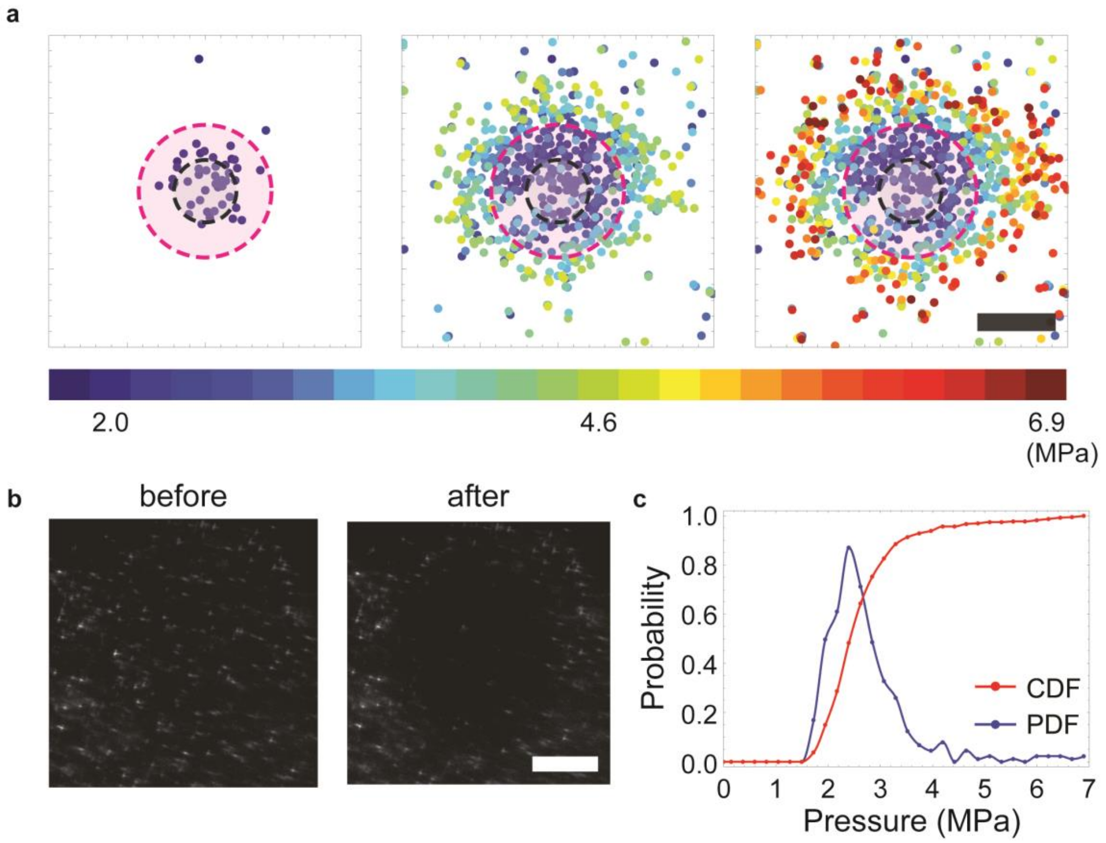

Supplementary Figure 4 | Measuring the probability distribution of microbubble destruction over pressure. A microbubble sheet was placed at the focal plane of an ultrasound beam. We directly observed the destruction of microbubbles with a camera and counted the number of destructed microbubbles at different pressures. (a) Distribution of destructed microbubbles over pressure. The positions of the destructed microbubbles were extracted using a watershed algorithm (see Methods). The big circle indicates the FWHM diameter of the ultrasound beam (~85 µm). We counted only the microbubbles within the small circle (40 µm), within which the pressure was approximated to be uniform. (b) Dark field images of the microbubbles before and after insonation with a sequence of pressures. (c) Resulting probability density function (PDF) and cumulative distribution function (CDF) of the microbubble destruction over pressure. Scale bars: 50 µm.

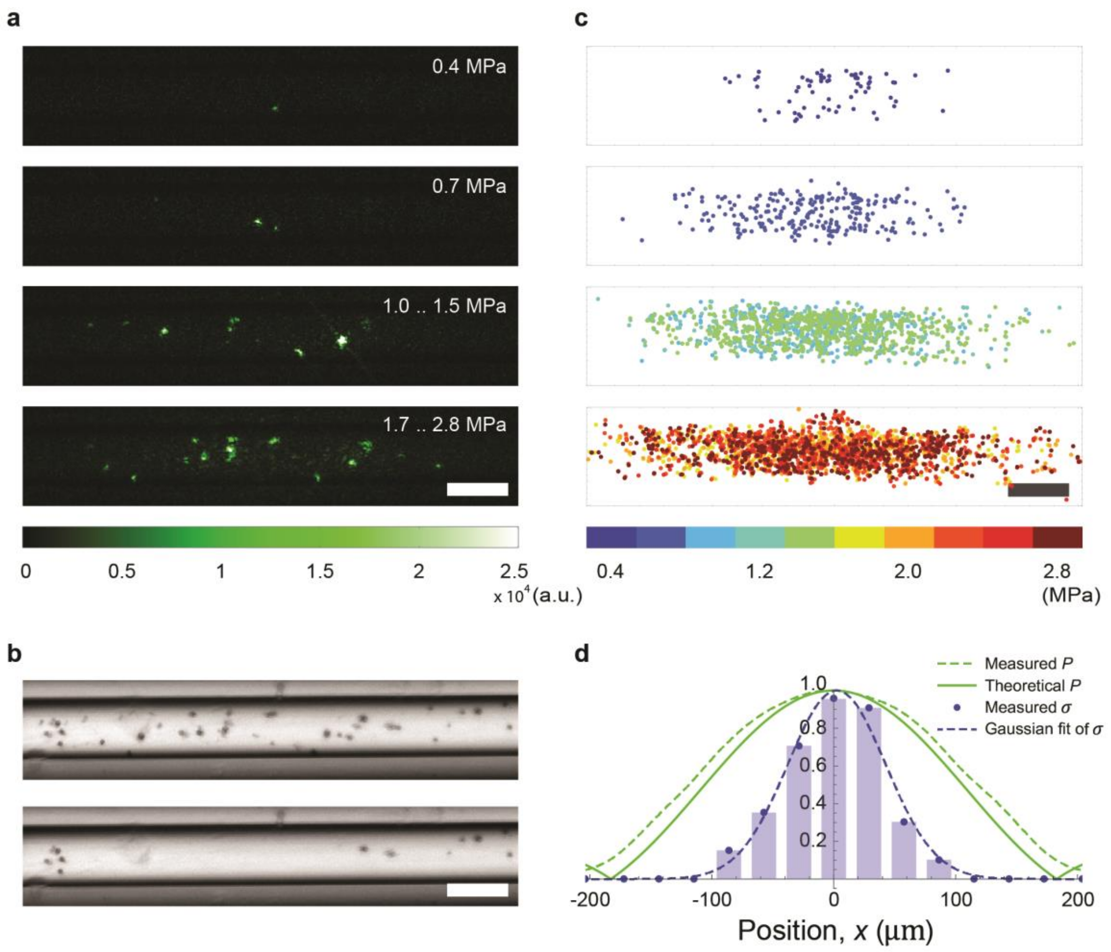

Supplementary Figure 5 | Exploring TRUME nonlinearity with 20 MHz ultrasound beam. Only lateral resolution was demonstrated because the axial focal zone of the 20 MHz ultrasound beam (2.3 mm, -6dB) exceeds the field of view of our observation system. We secured the microbubbles in agarose gel in an acrylic tube following the same protocol as described in the Methods. In order to demonstrate the relationship between lateral confinement of the TRUME foci and ultrasound pressure, we implemented TRUME to the same sample with 10 levels of pressures (linearly from 0.6 to 2.8 MPa) in ascending order. 91 sets of data were captured by targeting at different position of the sample. The TRUME foci were captured and processed in the same way as described the main article. (a) Images of TRUME foci at different ultrasound pressure groups from a typical set of data. (b) Corresponding microbubble images before and after microbubble destruction. (c) TRUME focus position distribution based on 91 sets of data, including that shown in (a). (d) Comparison of the histogram profile (FWHM of the fitted Gaussian profile: 95 μm) of the lower pressure group and the ultrasound lateral pressure profile (FWHM: 210 μm). Scale bars 50 μm.

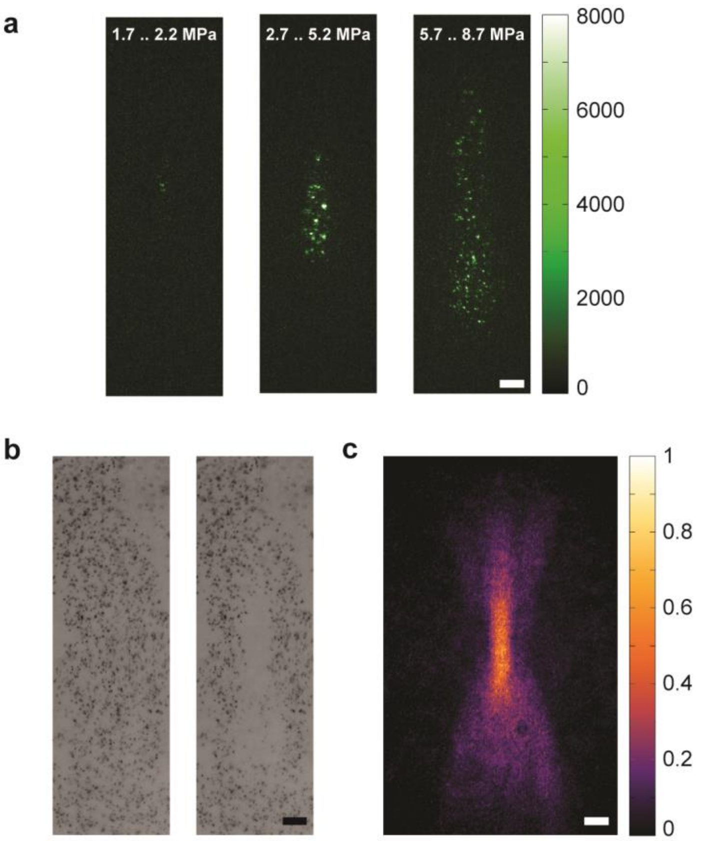

Supplementary Figure 6 | Raw images of the TRUME nonlinearity experiment (Figure 5b in the main article) and the corresponding optical image of the ultrasound beam. (a) Example images of the TRUME foci, which is one of the 135 sets of images that contribute to Figure 5b in the main article. In this case, we classified the images captured with 15 pressure levels into 3 pressure groups as indicated on the top of each figure and then averaged the images within the same group. (b) The corresponding microbubble images before and after insonation with a sequence of pressures. (c) The optical image of the ultrasound beam obtained by using digital holography. In this case, we imaged the ultrasound beam to the observation camera in the observation module (Supplementary Fig. 1) and acquired the optical field information by interfering with an extra reference beam (not shown in Supplementary Fig. 1). Scale bars: 50 µm.

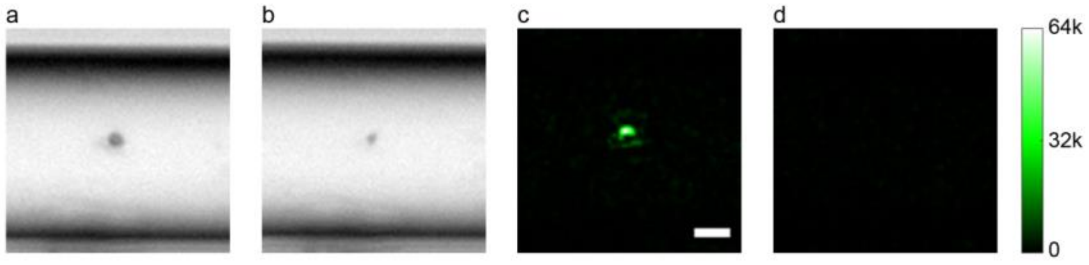

Supplementary Figure 7 | Optical focusing on size decreased microbubble. (a) Image of the microbubble before applying ultrasound. (b) After applying ultrasound. (c) Light focusing on the shrunk microbubble. (d) The focus vanished as the SLM phase pattern shifted by 10 pixels in both horizontal and vertical directions. Scale bar: 10 µm.

Supplementary Note 1 | Focusing on size-decreased microbubble.

The mechanisms of microbubble destruction include fragmentation and diffusion. While the fragmentation occurs within the timescale of microseconds, the acoustically driven diffusion effect takes up to tens of milliseconds[1]. Fundamentally, lower ultrasound pressure and larger microbubble diameter result in a longer time for the gas dissolution in the surrounding medium. In our experiment, we found that acoustically driven diffusion of microbubble gas takes longer in gel than in saline (PBS). Ideally for TRUME, the microbubbles are destroyed within the ultrasound duration (~29 ms in our experiment) as shown in Supplementary Movie 1 (scale bar: 10 µm). However, we also observed incomplete destruction of microbubbles within that period (Supplementary Movie 2), which typically occurs with low ultrasound pressure and large microbubble size. Nevertheless, this effect also enables an optical focus (Supplementary Fig. 7) to be formed. Alternatively, a longer insonation time allows the microbubble gas to completely dissolute into the surrounding medium (Supplementary Movie 3).

Supplementary Methods

Optical setup

The optical setup schematic diagram is shown in Supplementary Fig. 1. The setup can be partitioned into four major modules: light source regulation, DOPC, sample operation, and observation.

The light source regulation module provides three laser beams: a sample beam, reference beam, and playback beam. The intensity of each laser beam is regulated by a waveplate and a polarizing beam splitter. Both the sample beam and the reference beam are frequency-shifted by 50 MHz using acousto-optic modulators (AOMs). During hologram recording, the signal to AOM1 is phase-shifted following the trigger signals to the camera of the DOPC system. A path length matching arm is applied to the reference beam, as our laser has a relative short coherent length (~7 mm). All beams are spatially filtered using single mode fibers and collimated for DOPC application. The polarization of all beams is set to the horizontal direction, matching the polarization of the SLM. During DOPC recording, the shutter in the playback beam path (SH1) is closed and shutter in the sample beam path (SH2) is open. Both shutters flip after recording.

The DOPC module consists of a sCMOS camera (CAMS) and a SLM. These two components are precisely aligned[2] through a plate beam splitter (BSP) so that the optical field recorded by the camera can be reconstructed faithfully in space by the SLM. A polarizer is used to match the polarization of the sample beam and SLM. The sample beam and reference beam are combined through a 90% beam splitter (BST). The recorded phase of the sample beam is conjugated and sent to the SLM, which modulates the collimated playback beam. A beam compensator is used to compensate for the wavefront distortion introduced by the plate beam splitter.

In the sample operation module, light transmitted through the sample is collected by a 50 mm lens (L4) whose focus is positioned in the middle of the sample. An aperture (AP3) is

used to control the speckle area, which covers ~9 pixels in our case. A stage positioned ultrasound transducer is placed above the sample (normal to the diagram) but it is flattened to the left hand side in this two-dimensional diagram. The mirror (M4) for fluorescence signal detection is placed below the sample but also flattened in this diagram.

The observation module has an imaging system and a fluorescence detection system. In the imaging system, a ×20 objective (OB) and a 100 mm tube lens (L6) are arranged in a 4-f configuration through which the microbubbles in the sample are imaged onto a camera (CAM). In the experiment that demonstrates cytometry, the fluorescence emitted from the sample is collected by a lens (L5) and filtered by a fluorescence filter (FF). A single photon counting avalanche photodiode (APD) is used to detect the fluorescence signals.

Electrical signal flow for TRUE

The TRUE optical focus shown in Figure 2e was created using the same setup but with a modified signal flow (not shown in Supplementary Fig. 2). Instead of modulating the sample beam with an AOM, TRUE used single cycle ultrasound to shift the frequency of the sample beam so that the ultrasound encoded light was measured. The reference beam was modulated at 50.010 MHz rather than 50 MHz so that the camera averaged out the pattern due to interference between unmodulated light and reference beam, which was otherwise locked by the 20 kHz laser pulses. In this case, each ultrasound pulse was phase-inversed from the preceding pulse so that only ultrasound modulated light is locked by the reference beam[3]. The field measurement for TRUE was the same as that in TRUME.

PBR measurement

PBR was calculated from a region of interest (ROI, 600 pixels (x) by 40 pixels (y)) centred on the tube for both TRUME and TRUE foci. First, a one dimensional focus profile was extracted from the ROIs for peak calculation. For the TRUME focus, the row across the center of the focus was used as the one-dimensional focus. For the TRUE focus (Figure 2e), the one-dimensional focus was calculated by averaging the ROI in the y direction. In the second step, a one-dimensional Gaussian profile was fitted to each one-dimensional focus profile. The amplitude of the fitted Gaussian profile was considered as the peak intensity. Finally, to calculate the background light intensity, the SLM was shifted by 10 pixels in both x and y directions after the focus was made and the background image (e.g. Figure 3d in the main article) captured. The background intensity was estimated by averaging the ROI on the background image. The PBR was then calculated by taking the ratio between peak intensity and background intensity.

Modulation efficiency measurement

To compare the modulation efficiency of guidestar used in TRUE and TRUME, we measured the light fields modulated by the ultrasound focus and microbubble in clear media respectively and calculated the modulation efficiency. The field images of the guidestars were captured using the camera of the DOPC system.

a. Ultrasound modulation

We measured the ultrasound modulated light field based on the lock-in scheme and 4-phase stepping holography used for the phase recording of a TRUE process (see Electrical signal

flow). Mathematically, the optical field on the sensor plane can be decomposed into a reference field $E_{\mathrm{r}}(k)$ and a signal field E_{s}, which can be further decomposed into a modulated field E_{m} and an unmodulated field E_{u}. k denotes the step number of the phase stepping method. The complex fields $E_{\mathrm{r}}(k)$, E_{m} and E_{u} are given by

$$\begin{cases} E_{\mathrm{r}}(k) = A_{\mathrm{r}} \exp\left\{-i\left[\left(\omega_0 + \omega_{\mathrm{a}}\right)t + \varphi_{\mathrm{r}} + k\left(\pi/2\right)\right]\right\} \\ E_{\mathrm{m}} = A_{\mathrm{m}} \exp\left\{-i\left[\left(\omega_0 + \omega_{\mathrm{a}}\right)t + \varphi_{\mathrm{m}}\right]\right\} \\ E_{\mathrm{u}} = A_{\mathrm{u}} \exp\left[-i\left(\omega_0 t + \varphi_{\mathrm{u}}\right)\right] \end{cases}, \tag{1}$$

where A and φ denote the amplitude and phase of each complex field with associated subscripts, ω_0 and ω_{a} are the frequency of the light and ultrasound respectively. It should be noted that the ultrasound is assumed to be continuous wave here for simplicity. The light intensity on the sensor plane at the kth stepping phase can then be expressed as

$$I(k) = \left|E_{\mathrm{r}}(k) + E_{\mathrm{s}}\right|^2 = \left|E_{\mathrm{r}}(k) + E_{\mathrm{m}} + E_{\mathrm{u}}\right|^2. \tag{2}$$

By substituting the field terms with the right side of Supplementary Equation (1), we can further expand Supplementary Equation (2) to

$$\begin{aligned} I(k) = {} & A_{\mathrm{r}}^2 + A_{\mathrm{m}}^2 + A_{\mathrm{u}}^2 + 2A_{\mathrm{r}}A_{\mathrm{m}} \cos\left(\varphi_{\mathrm{r}} - \varphi_{\mathrm{m}} + k\pi/2\right) \\ & +2A_{\mathrm{r}}A_{\mathrm{u}} \cos\left(\omega_{\mathrm{a}}t + \varphi_{\mathrm{r}} - \varphi_{\mathrm{u}} + k\pi/2\right) + +2A_{\mathrm{m}}A_{\mathrm{u}} \cos\left(\omega_{\mathrm{a}}t + \varphi_{\mathrm{m}} - \varphi_{\mathrm{u}} + k\pi/2\right). \end{aligned} \tag{3}$$

The AC term $E_{\mathrm{r}}(k)E_{\mathrm{u}}^*$ and $E_m E_u^*$ has an oscillation frequency of ω_{a}. As the exposure time of the sensor is much longer than the ultrasound period ($=2\pi/\omega_{\mathrm{a}}$), these are effectively averaged out to 0. Then, the intensity captured from the sensor can be approximated as

$$I(k) \approx A_{\mathrm{r}}^2 + A_{\mathrm{m}}^2 + A_{\mathrm{u}}^2 + 2A_{\mathrm{r}}A_{\mathrm{m}} \cos\left(\varphi_{\mathrm{r}} - \varphi_{\mathrm{m}} + k\pi/2\right). \tag{4}$$

With the four measured interference patterns (at $k = 0,\ 1,\ 2$ and 3), we are able to compute the modulated field which is optically amplified by the reference light field:

$$\begin{aligned} E_{\mathrm{c}} &= \left(I_0 - I_2\right) + i\left(I_1 - I_3\right) \\ &= 4A_{\mathrm{r}}A_{\mathrm{m}} \exp\left(-i\Delta\varphi\right) \end{aligned}, \tag{5}$$

where the relative phase difference $\Delta\varphi$ replaces $\varphi_{\mathrm{r}} - \varphi_{\mathrm{m}}$. Then, by assuming the ideal modulation ($A_{\mathrm{m}} = A_{\mathrm{s}}$), the maximum possible amplitude of the computed field can be calculated:

$$\mathrm{Max}\left(\left|E_{\mathrm{c}}\right|\right) = 4A_{\mathrm{r}}A_{\mathrm{s}}. \tag{6}$$

Finally, we define the modulation efficiency

$$M \equiv \left(\frac{|E_\mathrm{c}|}{\mathrm{Max}(|E_\mathrm{c}|)} \right)^2 = \frac{|E_\mathrm{c}|^2}{16 I_\mathrm{r} I_\mathrm{s}}, \tag{7}$$

where the I_r and I_s are the reference (A_r^2) and signal beam intensity (A_s^2) respectively. I_r and I_s were measured separately to calculate the modulation efficiency. The modulation efficiency is then averaged over the ROI, whose diameter is the full width at half maximum (FWHM) of the one-dimensional modulation efficiency profile in the horizontal direction (Supplementary Fig. 3). In this experiment, the ultrasound peak pressure is ~2 MPa, and the calculated modulation efficiency is ~0.5%.

b. Microbubble modulation

We measured the microbubble modulated light field with phase stepping holography and field subtraction method used for the phase recording of a TRUME process. The reference field $E_\mathrm{r}(k)$, signal field before modulation $E_\mathrm{s}^{'}$ and signal field after modulation E_s can be expressed as

$$\begin{cases} E_\mathrm{r}(k) = A_\mathrm{r} \exp\left\{-i\left[\omega_0 t + \varphi_\mathrm{r} + k(\pi/2)\right]\right\} \\ E_\mathrm{s}^{'} = a_\mathrm{m} A_\mathrm{s} \exp\left\{-i\left[\omega_0 t + \varphi_\mathrm{s} + \Delta\phi\right]\right\} \\ E_\mathrm{s} = A_\mathrm{s} \exp\left\{-i\left[\omega_0 t + \varphi_\mathrm{s}\right]\right\} \end{cases}, \tag{8}$$

where the prime (') symbol denotes the signal field before modulation. Here we assume that the microbubble modulates the amplitude and phase of signal field by a_m (ranging from 0 to 1) and $\Delta\phi$ respectively. Then, the light intensity at the kth stepping phase is given by

$$\begin{cases} \text{before: } I^{'}(k) = \left|E_\mathrm{r}(k) + E_\mathrm{s}^{'}\right|^2 \\ \text{after: } I(k) = \left|E_\mathrm{r}(k) + E_\mathrm{s}\right| \end{cases}. \tag{9}$$

By substituting the field terms with the right side of Supplementary Equation (8), we have

$$\begin{cases} \text{before: } I^{'}(k) = A_\mathrm{r}^2 + a_\mathrm{m}^2 A_\mathrm{s}^2 + 2a_\mathrm{m} A_\mathrm{r} A_\mathrm{s} \cos(\varphi_\mathrm{r} - \varphi_\mathrm{s} - \Delta\phi + k\pi/2) \\ \text{after: } I(k) = A_\mathrm{r}^2 + A_\mathrm{s}^2 + 2A_\mathrm{r} A_\mathrm{s} \cos(\varphi_\mathrm{r} - \varphi_\mathrm{s} + k\pi/2) \end{cases}. \tag{10}$$

With the four measured interference patterns for each field measurement, we compute the signal fields (amplified by the reference field):

$$\begin{cases} \text{before: } E_\mathrm{c}^{'} = 4a_\mathrm{m} A_\mathrm{r} A_\mathrm{s} \exp\left[-i(\varphi_\mathrm{r} - \varphi_\mathrm{s} - \Delta\phi)\right] \\ \text{after: } E_\mathrm{c} = 4A_\mathrm{r} A_\mathrm{s} \exp\left[-i(\varphi_\mathrm{r} - \varphi_\mathrm{s})\right] \end{cases}. \tag{11}$$

Following the field subtraction method used in the TRUME process, we are able to calculate the amplitude of the modulated field $E_\mathrm{c}^{'} - E_\mathrm{c}$ (amplified by the reference field):

$$\left|E_\mathrm{c}^{'} - E_\mathrm{c}\right| = 4A_\mathrm{r} A_\mathrm{s} \left|1 - a_\mathrm{m} \exp(i\Delta\phi)\right|. \tag{12}$$

Maximum possible amplitude is achieved when $a_\mathrm{m} = 1$ and $\Delta\phi = \pi$:

$$\mathrm{Max}\left(\left|E_\mathrm{c}^{'} - E_\mathrm{c}\right|\right) = 8A_\mathrm{r}A_\mathrm{s} \ , \quad (13)$$

which is twice of the maximum possible amplitude for TRUE process (see Supplementary Equation (6)). This originates from the nature of field subtraction method involving two light field measurements. Finally, we define the modulation efficiency

$$M \equiv \left(\frac{\left|E_\mathrm{c}^{'} - E_\mathrm{c}\right|}{\mathrm{Max}(\left|E_\mathrm{c}^{'} - E_\mathrm{c}\right|)}\right)^2 = \frac{\left|E_\mathrm{c}^{'} - E_\mathrm{c}\right|^2}{64I_\mathrm{r}I_\mathrm{s}} \ . \quad (14)$$

We again measured the reference beam intensity I_r and signal beam intensity I_s separately and calculated the modulation efficiency. Similar to the ultrasound modulation case, the modulation efficiency is averaged over the ROI as shown Supplementary Fig. 3. The diameter of the ROI is again the FHWM of the one-dimensional modulation profile in the horizontal direction.

Microbubble destruction probability distribution

To characterise the microbubble destruction probability over pressure, we directly counted the number of destructed microbubbles at 31 discrete pressure levels (linearly from 0.15 to 6.88 MPa). It should be noted that TRUME was not implemented here, as the goal of this method was to count the number of microbubble destruction events. In this case, we designed a microbubble sheet (see Methods) and placed it at the focal plane of the ultrasound beam. In order to parallelize the measurement, we used an ultrasound beam with a wider pressure profile, which was generated by a lower numeric-aperture transducer (12.7 mm element diameter, 12.7 mm focal length, 50 MHz nominal central frequency, PI-50, Olympus). The transducer was driven by an ultrasound burst signal that had the same frequency (45 MHz), number of cycles (10) and interval time (10 µs) as the signal for the V3330 transducer.

An image of the microbubble sheet was captured after each insonation to the microbubbles, resulting in a set of 31 images at a single location. We repeated this process 95 times by targeting a new region and captured 95 sets of data. A watershed based algorithm (see Methods) was again used to extract the positions of the destructed microbubbles as shown in Supplementary Fig. 4a, and all the data sets were accumulated and classified into three pressure groups. The large circle (in magenta) outlines the FWHM contour of the pressure (diameter: ~85 µm), but we counted only those within the small circle (diameter: 40 µm), where the pressure was approximately uniform. Supplementary Fig. 4b shows typical images of the microbubbles before and after insonation with a sequence of pressures. Contrary to the microbubble images shown in the main article, here the images were captured in dark field because the ultrasound beam was normal to the microbubble sheet and oblique illumination was used. Supplementary Fig. 4c shows the probability density function (PDF) and cumulative distribution function (CDF) of the microbubble destruction over pressure.

Supplementary References

1. Chomas, J. E., Dayton, P., Allen, J., Morgan, K. & Ferrara, K. W. Mechanisms of contrast agent destruction. *IEEE Trans. Ultrason. Ferroelectr. Freq. Control* **48,** 232–48 (2001).

2. Jang, M., Ruan, H., Zhou, H., Judkewitz, B. & Yang, C. Method for auto-alignment of digital optical phase conjugation systems based on digital propagation. *Opt. Express* **22,** 14054–71 (2014).

3. Ruan, H., Mather, M. L. & Morgan, S. P. Pulsed ultrasound modulated optical tomography with harmonic lock-in holography detection. *J. Opt. Soc. Am. A. Opt. Image Sci. Vis.* **30,** 1409–16 (2013).
